# nc‐RNA‐mediated high expression of CDK6 correlates with poor prognosis and immune infiltration in pancreatic cancer

**DOI:** 10.1002/cam4.5260

**Published:** 2022-12-01

**Authors:** Yu‐Xuan Zhao, Bo‐Wen Xu, Fang‐Qing Wang, Feng‐Yang Jiang, Jian‐Wei Xu, De‐Xin Yu

**Affiliations:** ^1^ Department of Radiology, Qilu Hospital Cheeloo College of Medicine, Shandong University Jinan China; ^2^ Department of Hepatobiliary Surgery, Qilu Hospital Cheeloo College of Medicine, Shandong University Jinan China; ^3^ Department of Pancreatic Surgery, General Surgery, Qilu Hospital, Cheeloo College of Medicine Shandong University Jinan China

**Keywords:** CDK6, HOXA11‐AS, NR2F1‐AS1, pancreatic cancer, tumor immune infiltration

## Abstract

**Background:**

Emerging evidence manifests that cyclin‐dependent kinase 6 (CDK6) plays an essential part in the initiation and progression of several types of human cancer, and its descending expression is correlated with an adverse prognosis. However, the precise role of CDK6 in Pancreatic cancer (PC) remains obscure.

**Aims:**

To identify the potential ceRNA regulatory axis of CDK6 in PC and explore its relationship with immune cells and immune checkpoints.

**Materials & Methods:**

Using The Cancer Genome Atlas TCGA and GTEx data analyze the expression and survival of CDK6 in patients in pan‐cancer, and cellular experiments were performed to verify the effect of CDK6 on cell function. Using GEPIA and STARBASE databases to analyze prognosis, expression and survival, and identify non coding RNA (ncRNA) that mediates CDK6 overexpression. The TIMER 2.0 database was used for immune correlation analysis.

**Results:**

We revealed CDK6 might be an oncogene in PC, and the HOXA11‐AS /NR2F1‐AS1‐ miR‐454‐3p axis was identified as the possible upstream ncRNA‐associated pathway of CDK6 in PC. In addition, CDK6 show significant association with three immune checkpoints (PD‐L1, PD‐L2, and HAVCR2), the infiltration level of immune cells, and immunity biomarkers.

**Discussion:**

We discussed some applications of CDK6 in breast cancer, melanoma, and hemorrhagic malignancies. The role of miR‐15a‐5p, HOXA11‐AS and NR2F1‐AS1 in tumor development was also discussed based on existing studies. The potential mechanism of CDK6 affecting immune cells in pancreatic cancer was discussed.

**Conclusions:**

Overall, these results established that nc‐RNA‐mediated high expression of CDK6 is associated with patient outcomes and immune invasion in pancreatic cancer.

## 1 INTRODUCTION

Pancreatic cancer (PC) is one of the most lethal and aggressive malignancies tumors in humans, with a high mortality rate.^1^ Surgical resection for pancreatic cancer is the only alternative chance for curability, but it can only be achieved in early stage.^2^ However, most patients are already at an advanced stage when diagnosis is confirmed, and only 15%–20% of patients have the chance to surgery. Even if radical surgical resection, survival rate is still very low, with a 5‐year survival rate of only 2.9%.^3^ Therefore, it is important to identify key molecular targets or to seek prognostic biomarkers for promising PC.

Over the past decade, cyclin‐dependent kinase 6 (CDK6) has received increasing attention as a target for cancer therapy. CDK6 is a typical cyclin‐dependent kinase, as well as a transcriptional regulator involved in cell proliferation and differentiation.^4^, ^5^ The disorder of cell cycle‐related genes can lead to the dysregulation of cell cycles, resulting in unlimited cell proliferation and ultimately the development of cancer. Recently, growing evidences have demonstrated that CDK6 is closely associated with a variety of human tumors, especially hematologic malignancies, breast cancer and melanoma.^6^ In addition, in combination with taxanes, sequential administration of CDK4/6 inhibitors prevented the proliferation of pancreatic ductal adenocarcinoma (PDAC) cells.^7^ Selective CDK4/6 inhibitors have also been reported to not only modulate the cell cycle of tumor cells to induce their arrest, but also to facilitate anti‐tumor immune responses.^8^ However, a comprehensive analysis of CDK6 in the expression, prognosis, mechanism and immune microenvironment of PC remains lacking.

In this research, we first analyzed the expression of CDK6 in pan‐cancer and explored its relationship with prognosis. Then, the regulation of the noncoding RNA on CDK6 in PC, including microRNAs and long noncoding RNAs, were further investigated. In a further step, we investigated the connection between CDK6 expression and immune infiltration. Moreover, experiments were performed to verify the predicted results. Altogether, our research provided further proof for the action of CDK6 in cancer progression, suggesting that nc‐RNA‐mediated high expression of CDK6 is associated with patient outcomes and immune invasion in pancreatic cancer.

## 2 MATERIALS AND METHODS

### 2.1 Pan‐cancer analysis of CDK6 expression and prognosis

Expression date for CDK6 in pan‐cancer were downloaded from the Genotype‐Tissue Expression (GTEx) portal (https://gtexport.org/home/) and The Cancer Genome Atlas (TCGA) database (https://genome‐cancer.ucsc.edu/).^9^, ^10^ Data from normal tissues in the TCGA and GTEx databases were combined and compared with tumor tissues in the TCGA database to analyze differences in CDK6 expression. The normalized data were transformed into log2, and differential expression analysis was executed using the R package limma, and the results were visualized using the R package ggplot2. The *p* < 0.05 was considered statistically significant.

The gene expression profiling interactive analysis (GEPIA) (http://gepia.cancer‐pku.cn/) is an interactive web‐based database that enables normalized analysis of RNA‐Seq data from TCGA and GTEx databases.^11^ We used the GEPIA to predict the prognostic value of CDK6 in different tumor types and to evaluate the expression correlation of CDK6 with immune checkpoints in PC. The expression correlation of the Sankey diagram was constructed based on the R ggalluval package. In addition, the images of immunohistochemistry were downloaded from The Human Protein Atlas (THPA) (https://www.proteinatlas.org/) to visualize the expression and localization of CDK6 in tissues.

The clinicopathological features of patients with PC (including age, sex, race, grade, treatments, TNM stage, and gene) were subjected to univariate and multivariate Cox regression analysis by using the “survival” and “survminer” packages in R language.

### 2.2 Cell culture and transfections

Human pancreatic cancer cell line PANC‐1, MIA PaCa‐2, and AsPC‐1 were obtained from ATCC (Rockville, MD, USA). They were cultured in high glucose Dulbecco's modified eagle medium (DMEM) (Gibco, Beijing, China) with 10% fetal bovine serum (FBS) (Gibco, Beijing, China) in 37°C and 5% CO_2_ incubators. The siRNA was transfected into human pancreatic cancer cells with Micropoly transfecter TM cell reagent (Micropoly, China) as the manufacturer's recommendation. Target sequences for siRNAs were as follows: Sense, 5′‐GUUUGAACAUGUCGAUCAATT‐3′ and anti‐sense, 5′‐UUGAUCGACAUGUUCAAACTT‐3′ (CDK6 si1); Sense, 5′‐GAGAAGUUUGUAACAGAUATT‐3′ and anti‐sense, 5′‐UAUCUGUUACAAACUUCUCTT‐3′ (CDK6 si2); Sense, 5′‐AAGUUCAGAUGUUGAUCAATT‐3′ and anti‐sense, 5′‐UUGAUCAACAUCUGAACUUTT‐3′ (CDK6 si3).

### 2.3 RNA extraction, RT‐PCR, and qRT‐PCR

Total RNA was extracted from PANC‐1, MIA PaCa‐2, and AsPC‐1 with the SPARKeasy Cell RNA Kit (Sparkjade) and then reverse transcription was performed according to the manual of NovoScript® 1st Strand cDNA Synthesis SuperMix (Novoprotein Scientific Inc. China). Next, RT‐qPCR reactions were prepared with the NovoStart®SYBR qPCR SuperMix Plus (Novoprotein, Nanjing, China) with U6 as the internal reference of miR‐454‐3p and GAPDH as the internal reference of NR2F1‐AS1, CDK6 and CDK4. Primers were shown as follows: GAPDH, Forward (F): 5′‐GCACCGTCAAGGCTGAGAAC‐3′, Reverse (R): 5′‐TG GTGAAGACGCCAGTGGA‐3′. U6: Forward (F): 5′‐CTCG CTTCGGCAGCACA‐3′, Reverse (R): 5′‐AACGCTTCACG AAYYYGCGT‐3′.CDK4, Forward (F): 5′‐ATGGCTACCT CTCGATATGAGC‐3′, Reverse (R): 5′‐CATTGGGGACTC TCACACTCT‐3′.CDK6, Forward (F): 5′‐TCTTCATTCAC ACCGAGTAGTGC‐3′, Reverse (R): 5′‐TGAGGTTAGAGC CATCTGGAAA‐3′.

### 2.4 The scratch‐wound assay

Transfected cells were seeded into six‐well plate at a density of 1 × 10^5^ cells per well and maintained in a humidified incubator with 37 C, 5% CO_2_. A 1000‐μl pipette tip was used to obtain the cell free lane. The cells were washed three times using PBS to clear detached cells, followed by incubation with DMEM without FBS at 37°C. Images were taken using a X71 (U‐RFL‐T) fluorescence microscope (Olympus, Melville, NY).

### 2.5 Transwell invasion and migration assay

For transwell migration assay, 10 × 10^4^ cells were seeded in the upper chambers, and 700 μl DMEM containing 10% FBS was added to the lower chamber. After incubation at 37°C for 48 h, cells were washed using PBS and fixed with methanol at room temperature for 30 min. Next, cells were stained in 0.1% crystal violet at room temperature for 5 min and observed under a microscope. For transwell invasion assay, we precoated 1:8 ratio diluted matrigel (BD Biosciences, San Jose, CA) in the bottom of upper chambers of the transwell plates (8 μm, Corning Costar, MA, USA). Cells in serum‐free media were seeded onto the filter of upper chambers and added DMEM contained 10% FBS to lower chambers. Then incubated the plates for 72 h and observed under a microscope.

### 2.6 Screening of potential miRNAs of CDK6

We used several online target gene prediction software to predict miRNA that potentially regulates CDK6, consisting of starBase, miRWalk, mirRDB, and TargetScan, specifically the screened miRNAs were generated in more than three programs as mentioned above. The expression levels of candidate miRNAs in PC and their prognostic role on the overall survival of PC patients were further validated using starBase (http://starbase.sysu.edu.cn/).^12^

### 2.7 Potential lncRNAs of miRNAs

The lncRNAs that potentially regulating the screened miRNA were all predicted by the starBase and LncBase (http://carolina.imis.athena‐innovation.gr/) together. Using starBase to conduct correlation analysis on miRNAs‐CDK6, lncRNAs‐miRNAs, and lncRNA‐CDK6 expression in PC.

### 2.8 Relationship between CDK6 expression and immunity

We use tumor immune estimation resource (TIMER) database 2.0 (https://cistrome.shinyapps.io/timer/) to systematically analyze the correlation between CDK6 expression and infiltration of six major immune cells in PC.^13^ In addition, we explored the relevance of CDK6 expression to the level of immune checkpoint markers using the correlation module.^14^ The built‐in algorithm ssGSEA of R package GSVA^15^ was used to analyze the relationship between CDK6 expression and 24 immune cell biomarkers identified from a study published by Bindea et al.^16^ The analysis data come from the level 3 HTSeq‐FPKM RNA‐seq data in the PAAD project of TCGA.

### 2.9 Survival and prognosis analysis

The association of CDK6 expression with cancer prognosis was assessed using GEPIA. Median expression of miR‐454‐3p divided patients into different risk groups, and the effect of miR‐454‐3p on OS in PC was visualized by online tool Kaplan–Meier plotter (http://kmplot.com/analysis/).^17^

### 2.10 Statistical analysis

Pan‐cancer analysis was performed using the Kruskal–Wallis test, and we assessed CDK6 expression levels in 33 cancer tissues. Wilcox tests were used to assess CDK6 expression in patients with different pathological stages. Independent sample t‐tests were performed to verify the different expression levels of CDK6 in tumor and normal tissues. Survival curves (Kaplan–Meier) was used to demonstrate the survival of patients between groups. *p* < 0.05 or log rank *p* < 0.05 was considered as statistically significant.

SPSS (SPSS, Inc., Chicago, IL, USA) software was used to perform univariate analysis on all possible risk factors of PAAD. Variables with *p* < 0.3 in the analysis results were included in the multivariate analysis. The calculation method was input. Then, the variables with *p* ≥ 0.05 in the multivariate analysis were removed in order to observe the maximum Exp (B). If Exp (B) changes by more than 10% after removal, the variable is retained. If not, remove the variable.

## 3 RESULTS

### 3.1 Integrated analysis of the expression of CDK6 in pan‐cancer via TCGA and GTEx datasets

**FIGURE 1:**
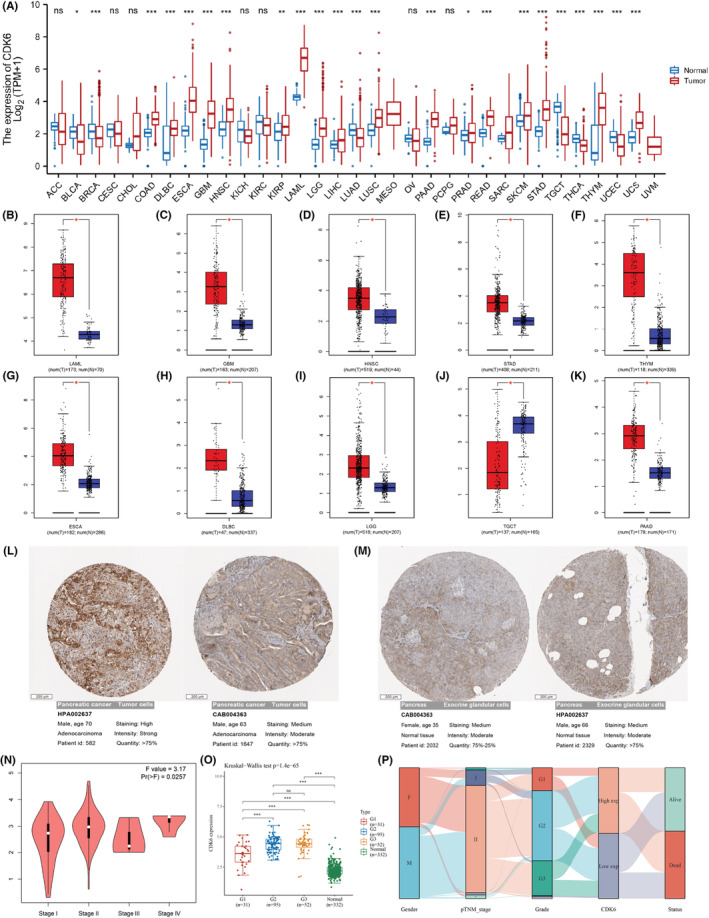
Expression level of CDK6 gene in multiple tumors and its relationship with pathological and clinical characteristics in PC patients. (A) CDK6 expression in 33 types of tumor and normal tissues based on the TCGA and GTEx database. *p < 0.05; **p < 0.01; ***p < 0.001. (B–K) Red, tumor tissue; blue, normal tissue. The expression of CDK6 in different cancer types based on the GEPIA database. *p < 0.05. (L) Immunohistochemical images and sample information of CDK6 expression in representative tumor tissues based on the THPA database. Two antibodies. (M) Immunohistochemical images and sample information of CDK6 expression in representative normal tissues based on the THPA database. Two antibodies. (N) Violin plot showing CDK6 expression in PAAD matched with TCGA normal data and GTEx data, results based on GEPIA. (O) The expression of CDK6 in different histologic stage patients. ***p < 0.001. (P) Sankey diagram showing CDK6 expression association with different clinical characteristic.

First, the expression of CDK6 in normal and cancer tissues were examined by integrating TCGA and GTEx datasets. The Figure 1A demonstrated that CDK6 was markedly up‐regulated in COAD, DLBC, ESCA, GBM, HNSC, KIRP, LAML, LGG, LIHC, LUSC, PAAD, PRAD, READ, SKCM, STAD, THYM, and UCS tumors, and was significantly down‐regulated in BLCA, BRCA, LUAD, TGCT, THCA, and UCEC. However, the expression of CDK6 exhibited no marked difference in ACC, CESC, CHOL, KICH, KIRC, OV, or PCPG.

Next, the GEPIA database was further used to validate our results. As described in Figure 1B–K, compared with normal tissues, CDK6 expression was significantly increased in 9 types of cancer, including DLBC, ESCA, GBM, HNSC, LAML, LGG, PAAD, STAD, and THYM. In addition, CDK6 expression in TGCT was statistically decreased when compared with corresponding TGCA normal controls.

**FIGURE 2:**
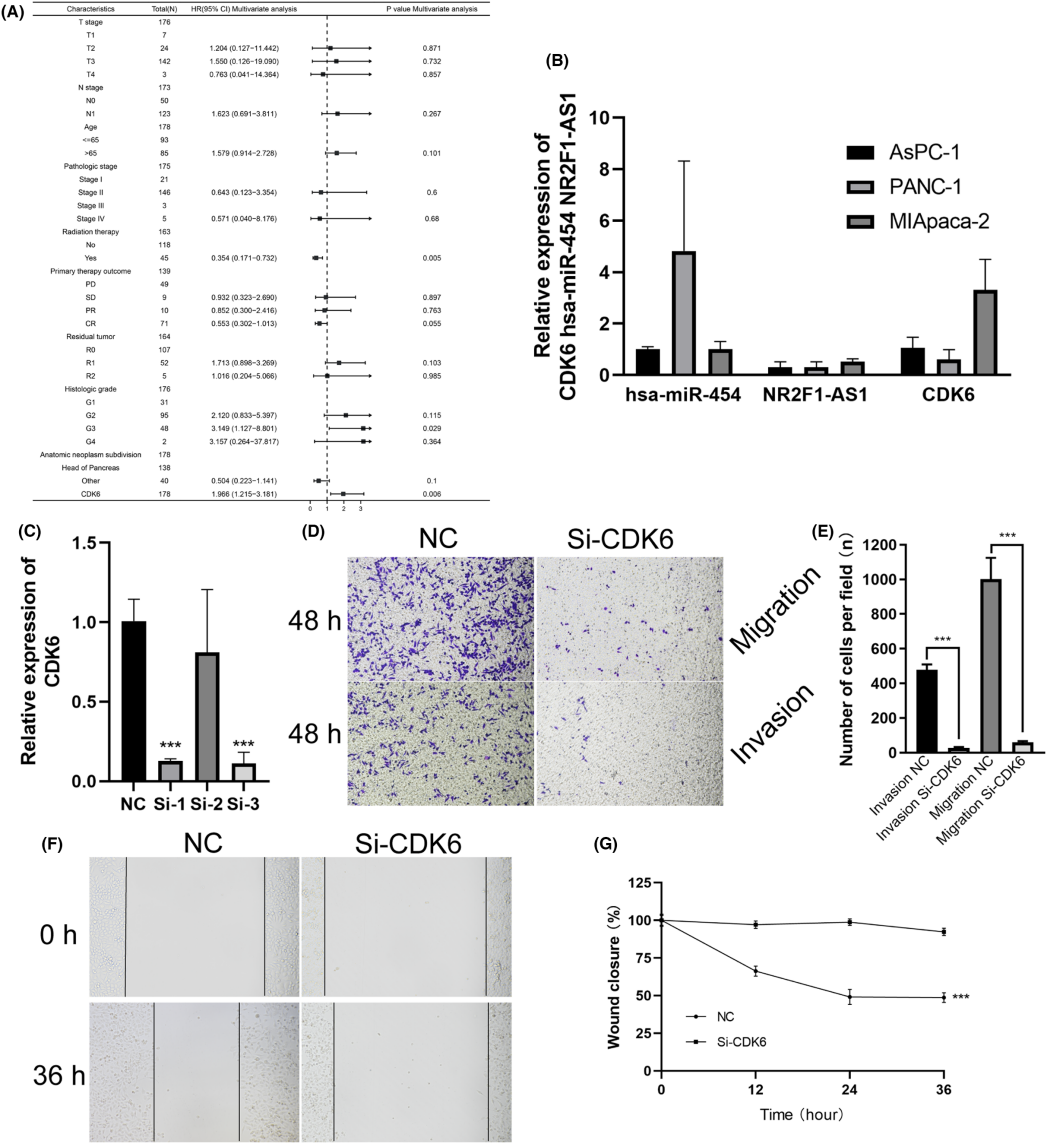
CDK6 knockdown inhibits the invasion and migration of pancreatic cancer. (A) CDK6 is an independent risk factor predicting the prognosis of PC patients based on TCGA data. Using R language to analyze relevant data. (B) The expression of CDK6, miR‐454‐3p, NR2F1‐AS1 in PANC‐1, MIA PaCa‐2, and AsPC‐1 cells were respectively detected by qRT‐PCR. (C) The expression of CDK6 mRNA in MIA PaCa‐2 cells after transfection cells with CDK6 short‐hairpin RNA (sh‐CDK6) and control short‐hairpin RNA (sh‐NC) was detected by qRT‐PCR. ***p < 0.001. (D) Representative images of transwell migration and invasion assays in MIA PaCa‐2 cells after transfection with sh‐CDK6 and sh‐NC. Magnification, ×200. scale bar, 50 μm. (E) Quantification of transwell migration and invasion assays. *n* = 2. ***p < 0.001. sh‐NC vs. sh‐CDK6 group. (F) Representative images of MIA PaCa‐2 cells after transfection with sh‐CDK6 and sh‐NC was measured by the scratch assay. Magnification, ×100; scale bar, 100 μm. (G) Quantification of the scratch assay. ***p < 0.001. sh‐NC vs. sh‐CDK6 group.

We used THPA database further queried the immunohistochemical staining results. The result showed that CDK6 expression was markedly higher in PC tumor tissues (Figure 1L,M). These results indicated that up‐regulated of CDK6 in PC predicted advanced malignancy. In the end, we assessed CDK6 expression in PC patients with different TNM stages, where CDK6 expression was higher in the grade III/grade IV group than in the grade I/grade II group (Figure 1N) (*p* < 0.05). In addition, CDK6 was also correlated with the histological stage of pancreatic cancer (Figure 1O) (*p* < 0.05). The Sankey diagrams showing the distribution trends of different CDK6 gene expression in diverse clinical features (Figure 1P). Univariate and multivariate analyses showed that high CDK6 expression was an independent risk factor for the prognosis of patients with PC (Figure 2A; Table S1).

### 3.2 High expression of CDK6 is associated with cell migration and invasion in PAAD

First, we explored the expression of CDK6 in PANC‐1, MIA PaCa‐2, and AsPC‐1 cells via RT‐qPCR and found CDK6 owned the highest expression in MIA PaCa‐2 cells (Figure 2B). In order to study the function of CDK6 in pancreatic cancer cells, the siRNA for silencing CDK6 were transfected into MIA PaCa‐2 cells. Following CDK6 knockdown, the relative mRNA expression of CDK6 was remarkably lower compared with the siNC group (Figure 2C). Additionally, the transwell invasion and migration assay showed that the cell invasion capacities were remarkably inhibited in CDK6‐silenced cells (Figure 2D,E), and the scratch assay showed that migration ability was impaired in CDK6‐silenced cells (Figure 2F,G) (***p < 0.001).

### 3.3 Prognostic analysis of CDK6 in pan‐cancer

**FIGURE 3:**
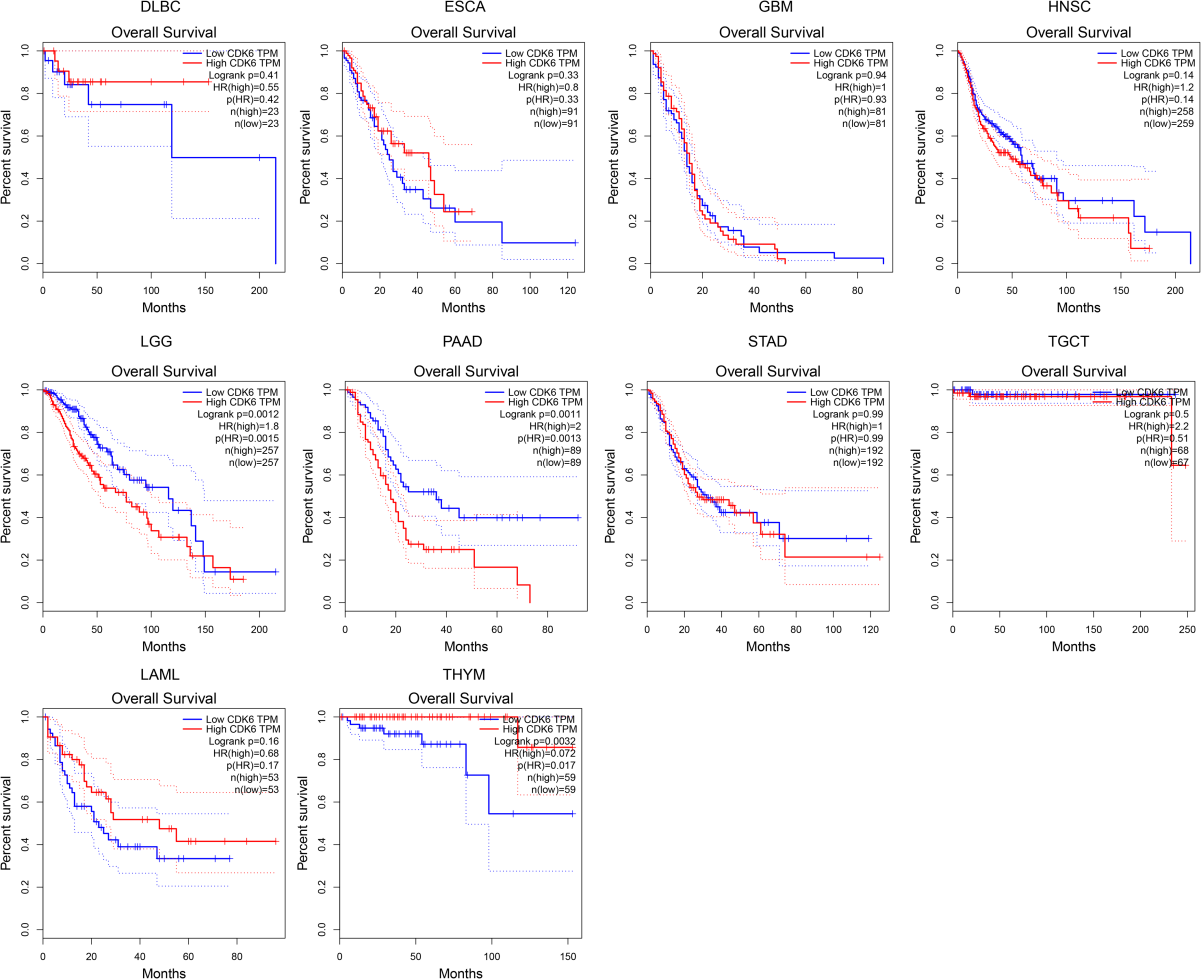
Analysis of the overall survival (OS) of CDK6 in 10 types of human cancers based on the GEPIA database.

We evaluated whether CDK6 expression was associated with OS and RFS in multiple cancer. The outcomes indicate that patients with high CDK6 expression in PAAD and LGG tend to suggest poorer OS (Figure 3). For RFS, CDK6 was a high‐risk factor in LGG and PAAD, while a protective factor in THYM (Figure S1).

In addition, since CDK6 and CDK4 are homologous proteins with similar physiological function, we further explored the expression of CDK4 in PC and its relationship with prognosis. The results showed that high expression of CDK4 was not correlated with poor prognosis of patients with PAAD (OS; *p* = 0.56, RFS; *p* = 0.52) (Figure S2).

### 3.4 Exploration of the possible regulation mechanisms of CDK6 in PC

**FIGURE 4:**
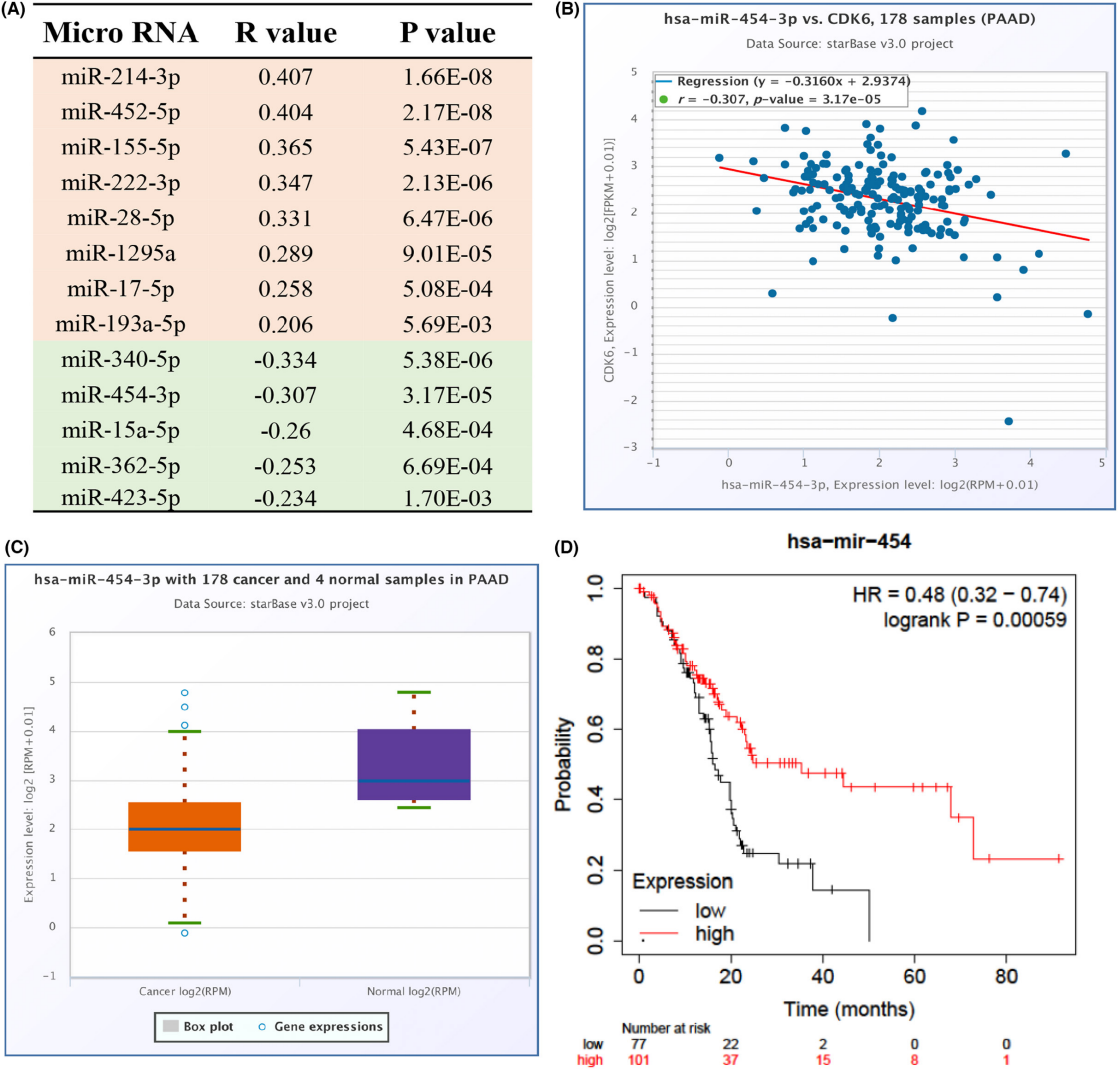
In PAAD miR‐454‐3p acts as a possible miRNA regulating CDK6. (A) Multiple databases jointly predict the correlation between miRNA and CDK6 expression in PAAD. Plot A shows the 13 miRNA (*p* < 0.01) with the largest relationship among all 48 relevant miRNA. (B) Association of CDK6 with miR‐454‐3p in PAAD retrieved from starBase database. (C) Expression of miR‐454‐3p in tumor and normal tissues in PAAD as shown by starBase database. (D) Kaplan–Meier curves revealed the influence of miR‐454‐3p expression on the prognosis of PC patients.

To investigate the possible regulation mechanisms of CDK6 in PC, we used multiple databases to explore the potential upstream nc‐RNAs of CDK6. First, we explored the potential upstream miRNAs of CDK6 and finally found 48 miRNAs (Table S1). In general, the expression of the miRNA target genes was negatively correlated with the expression of corresponding miRNA. Therefore, bioinformatics analyses were used to indicate the expression correlation analysis in PC and the relationship with prognosis. Figure 4A,B shows that the expression of CDK6 was significantly negatively correlated with miR‐340‐5p, miR‐454‐3p, miR‐15a‐5p, mitR‐362‐5p, miR‐423‐5p (*p* < 0.01), and positively correlated with miR‐214‐3p, miR‐452‐5p, miR‐155‐5p, miR‐222‐3p, miR‐28‐5p, miR‐1295a, miR‐17‐5p, miR‐193a‐5p in PC (*p* < 0.01). Relative data were downloaded from TCGA database and subjected to expression and survival analysis. Analysis results showed that miR‐454‐3p (Figure 4C) and miR‐15a‐5p (S1A) expression was decreased in pancreatic cancer (*p* < 0.01), and the low expression of miR‐454‐3p (Figure 4D) and miR‐15a‐5p (S1B) proposed an unfavorable prognosis in pancreatic cancer patients (*p* < 0.01). Through the combination of survival and expression analysis, miR‐454‐3p and miR‐15a‐5p were identified as a putative miRNA targeting CDK6.

### 3.5 Prediction of miR‐454‐3p associated lncRNAs

**FIGURE 5:**
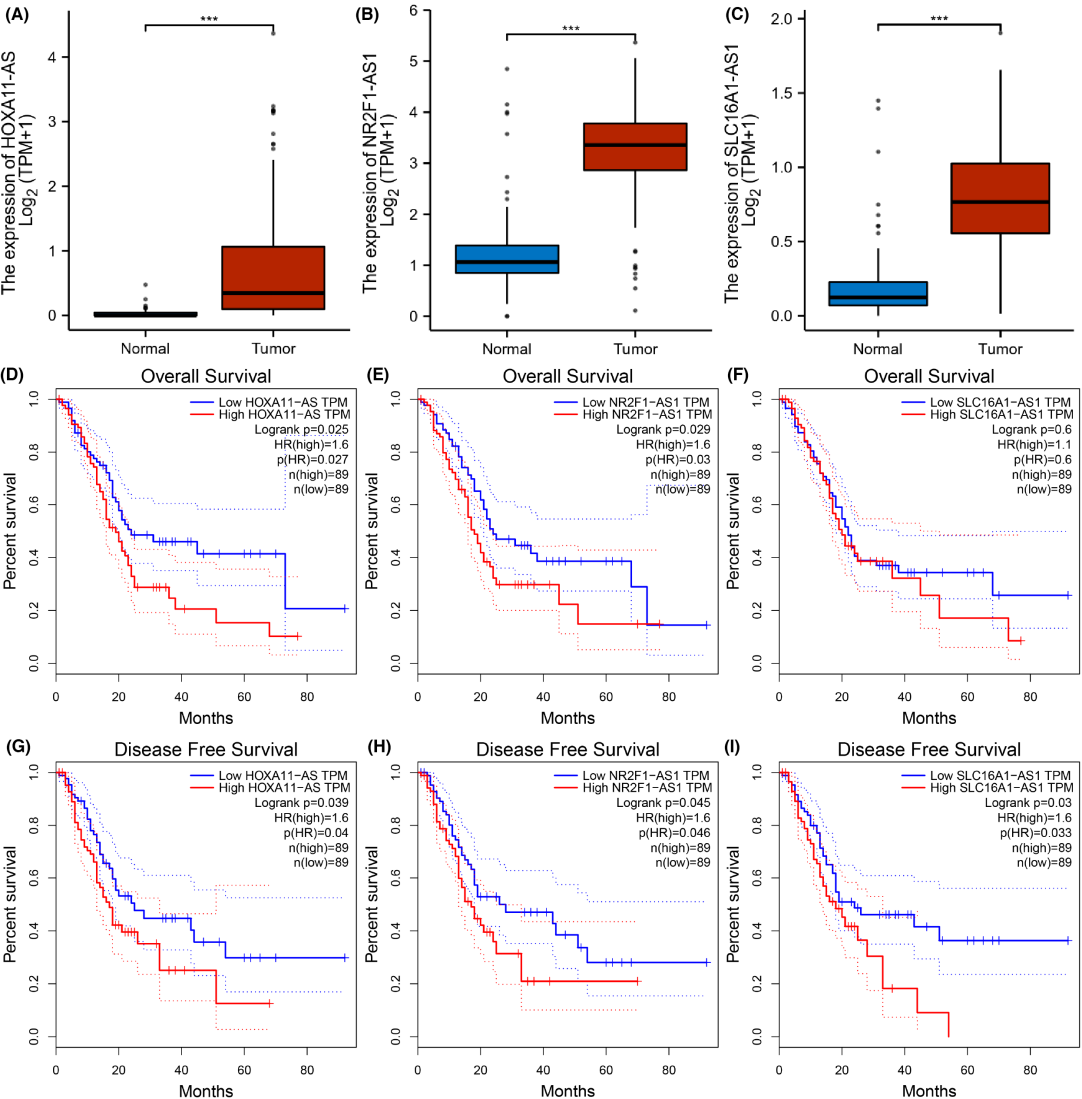
Expression and survival analysis of lncRNA regulating miR‐454‐3p in PAAD. (A–C) The expression of (A) HOXA11‐AS, (B) NR2F1‐AS1, and (C) SLC16A1‐AS1 in TCGA PAAD compared with “GTEx normal and TCGA” data. (D–F) The OS for (D) HOXA11‐AS, (E) NR2F1‐AS1, and (F) SLC16A1‐AS1 in PAAD. (G–I) The RFS for (G) HOXA11‐AS, (H) NR2F1‐AS1, and (I) SLC16A1‐AS1 in PAAD. ***p < 0.001

Due to the potential lncRNA of miR‐15a‐5p in starBase and LncBase was not followed the ceRNA regulation rules. Thus, only the candidate lncRNAs of miR‐454‐3p were identified through starBase and LncBase databases. A total of 123 possible lncRNAs were forecasted in starBase and 319 in the LncBase. Then we select 28 lncRNAs that have appeared in both databases for analysis (Table S2). For better visualization, lncRNAs‐miR‐454‐3p were constructed as presented in S2. Next, the expression and prognosis of these lncRNAs were analyzed using GEPIA. Among all the 28 lncRNAs, only H19, HCG18, NUTM2A‐AS1, NR2F1‐AS1, HOXA11‐AS, PRKCQ‐AS1, SLC16A1‐AS1, and AC005519.1 were significantly upregulated, and AL158206.1, HIPK1‐AS1, VASH1‐AS1, AC004812.2 were downregulated in PC. Besides, the higher expression of HOXA11‐AS and NR2F1‐AS1 significantly indicated both better OS and RFS, and overexpressed SLC16A1‐AS1 indicated better RFS of PC (Figure 5). Next, we explored the correlation between the expression levels of three lncRNAs and miR‐454‐3p or CDK6 in PC, as depicted in S3. By combination of survival analysis, expression analysis, and correlation analysis, HOXA11‐AS and NR2F1‐AS1 might be the regulatory lncRNA with most potential of the target miR‐454‐3p/CDK6 axis in PC.

### 3.6 Correlations between CDK6 and immune infiltration levels and immune checkpoint gene expression in PC

**FIGURE 6:**
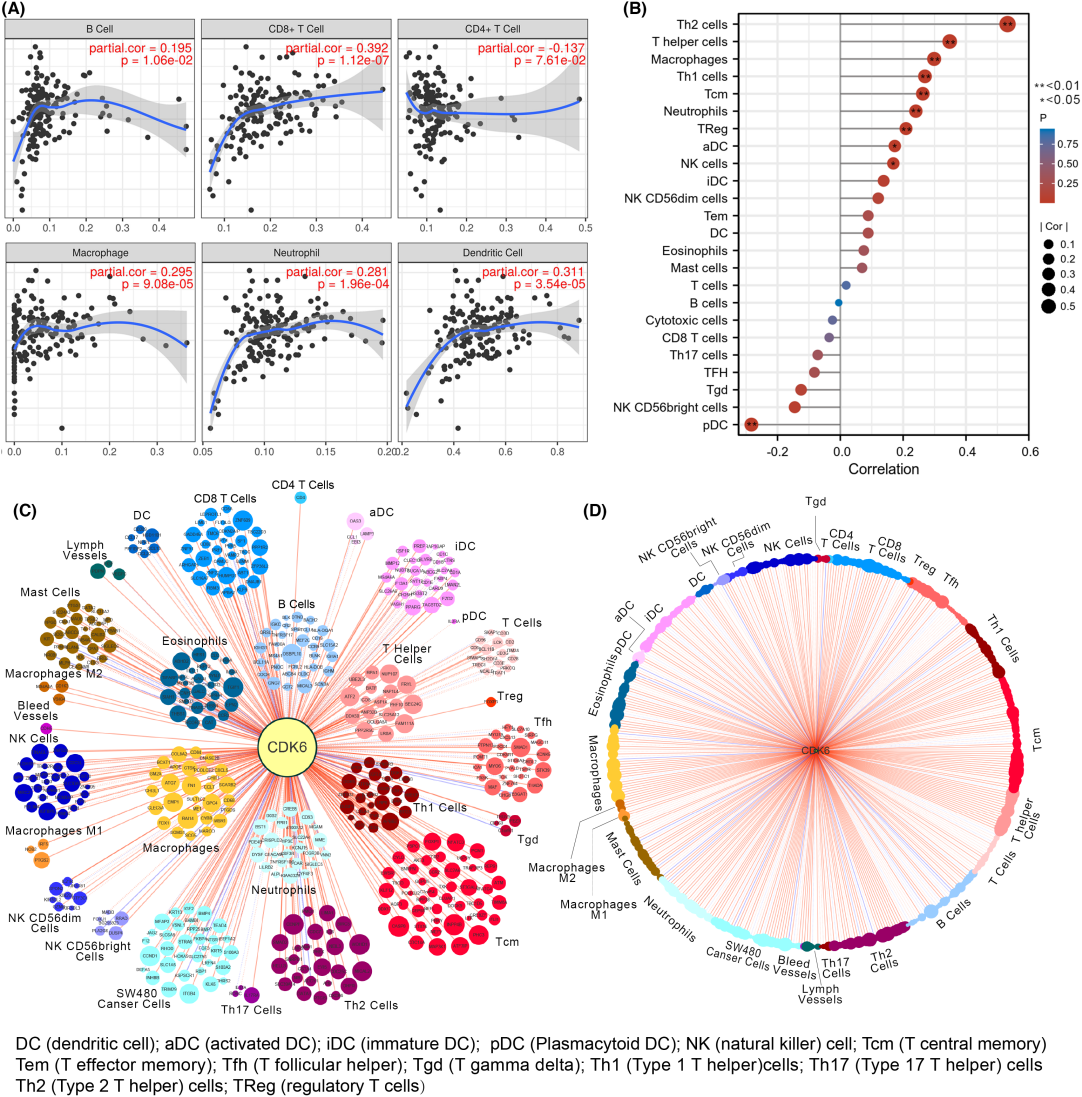
CDK6 in PAAD is associated with immune cell infiltration. (A) The correlation analysis of CDK6 level with 6 kinds of immune cells in PAAD. (B) The lollipop plot showed the correlation of 24 immune cell and its subtype infiltration with CDK6 in PAAD. (C) Cytoscape visualization the relationship between CDK6 and various immune cell biomarkers in PAAD. (D) Cytoscape visualization the correlation between 24 immune cell and CDK6 in PAAD determined by *R* (V 3.6.3). Dotted line: *p* > 0.05, Blue lines: *R* < 0, Red lines: *R* > 0. The color depth of the line increases with the decrease of *p* value, and the area of the colored dots indicates the absolute value of the *R*.

A growing number of studies have showed that CDK6 not only plays an essential part in the cell cycle, but also in immunosuppression of tumors. In certain tumors, CDK6 inhibitors can exert antitumor effects through immune activation. Consequently, we tried to find the relationship between CDK6 and immune cells by correlation analysis. First, the immune cell infiltration was obtained from the TIMER website. The expression level of CDK6 was found to be strongly correlated with multiple immune cells in PC as shown in Figure 6A.

To give clues to investigate the function and mechanism of CDK6, we further analyzed the correlation between 24 immune cells^16^ and CDK6 expression and visualized the results (Figure 6B). The results showed the high CDK6 expression was significantly correlated with pDC, NK cells, aDC, Treg, Neutrophils, Tcm, Th1 cells, Macrophages, T helper cells, Th2 cells. Also, we analyzed and visualized the relationship between CDK6 expression and these immune cell surface markers (Figure 6C,D). These results validate a certain degree of positive correlation between CDK6 levels and cell infiltration.

**FIGURE 7:**
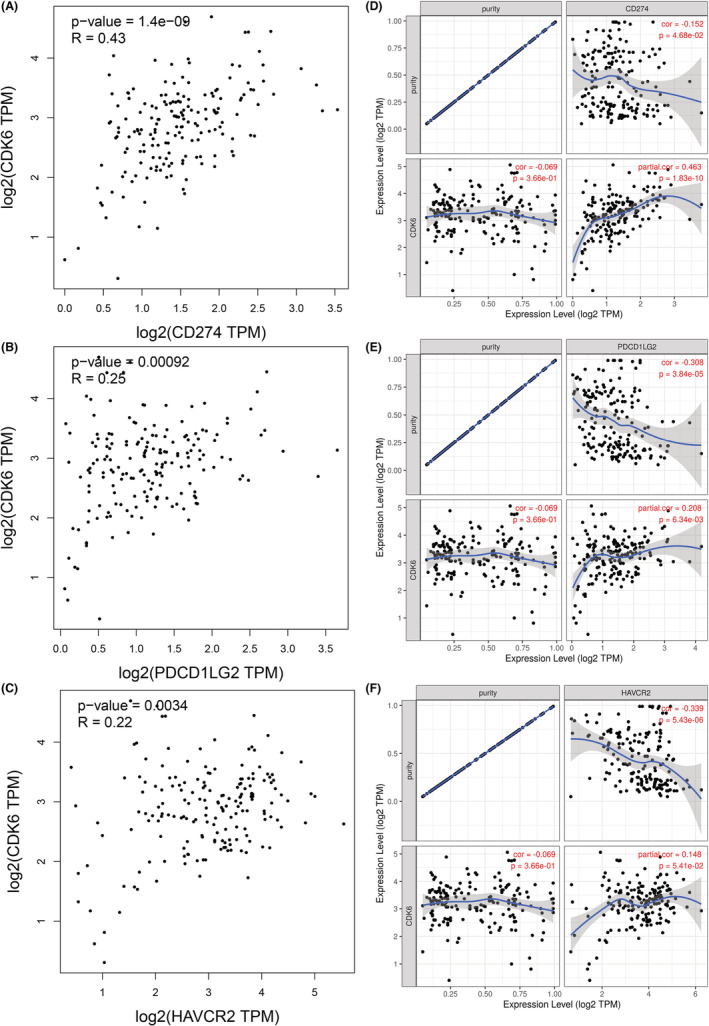
Relationship of CDK6 with PD‐L1, PD‐L2, and HAVCR2 (TIM‐3) expression in PAAD. (A–C) GEPIA database analysis the correlation between CDK6 expression and PD‐L1, PDCD1LG2 (PD‐L2), and HAVCR2 (TIM‐3) in PAAD. (D–F) Correlation between CDK6 and PD‐L1, PDCD1LG2 (PD‐L2), and HAVCR2 (TIM‐3) expression in PAAD.

Regarded as a novel immunotherapy agent, immune checkpoint inhibitors (ICIs) have considerably transformed clinical decision‐making in tumor immunotherapy. Considering the potential carcinogenic role of CDK6 in PC, we analyzed the correlation between CDK6 and the expression of eight classical immune checkpoint genes. Our analysis using the online database GEPIA demonstrated that CDK6 was significantly positively correlated with PDL1, PDL2 and HAVCR2 expression in PC (Figure 7A–C). To make the results more accurate, TIMER database was used to validate the results according to adjusting tumor purity. As a result, CDK6 was significantly correlated with PDL1 and PDL2 (Figure 7D–F). Overall, these findings supported that CDK6 plays an instrumental part in tumor immunity.

## 4 DISCUSSION

It is well known that there are many obstacles in the clinical, molecular, and biomedical research of PC. Therefore, exploring its underlying pathogenesis may provide potential targets and new therapeutic strategies for regulating tumor progression. Several studies have shown that some kinases founded in breast cancer, melanoma and hematopoietic malignancies are able to regulate the function of CDK6 and CDK4, which makes CDK6 an attractive target for therapeutic disruption.^6^ Salvador Barbero et al. found that sequential administration of CDK4/6 inhibitors were able to block the proliferation of paclitaxel‐treated pancreatic ductal adenocarcinoma (PDAC) cells.^7^ However, the knowledge of CDK6 still needs further investigation.

First, we performed pan‐cancer analysis of CDK6 with TCGA and GTEx databases, and then further validated CDK6 expression using the GEPIA. Furthermore, survival analysis of CDK6 in these cancer types revealed that high CDK6 expression was associated with a poorer prognosis in PC. A study by Cao et al. also confirmed that high expression of CDK6 promotes invasion and metastasis of pancreatic cancer cells.^18^ Taken together, these results suggest an oncogenic role for CDK6 in PC.

Many studies suggested that non‐coding RNA (ncRNAs), interact with each other through the ceRNA regulatory mechanism to participate in the regulation of gene expression.^19^, ^20^, ^21^ Extensive studies have also demonstrated that lncRNAs can regulate miRNAs and further interact with mRNA expression through the ceRNA mechanism. Notably, one miRNA can be regulated by multiple LncRNAs and one mRNA can be targeted by several miRNAs in PC. For instance, Zu et al. found PVT1/miR‐20b/CCND1 ceRNA regulatory network associated with PC progression by comprehensive analysis.^22^ Gao et al. demonstrated that lncRNA DLEU1 promotes the development of PDAC through the miR‐381/CXCR4 axis.^23^ However, to our knowledge, a complete ncRNA–miRNA regulatory axis associated with CDK6 has not been established in PC to date.

In our study, four prediction programs, including starBase, miRWalk, mirRDB, and TargetScan, were introduced to explore the upstream regulatory miRNAs of CDK6, and 48 miRNAs were finally obtained. After, we performed a differential expression analysis by using CDK6 miRNA information of starBase. In order to gain less noise and more reliable difference in the next step, we choose the miRNA with *p* < 0.001. At the end, 5 CDK6‐associated miRNAs consistent with ceRNA regulatory rules were identified. The expression and function of our screened miRNAs in PC were mostly consistent with previous findings. For instance, miR‐340‐5p inhibits growth and invasion of PAAD cells by targeting TGFA.^24^ By comprehensive analysis, miR‐454‐3p and miR‐15a‐5p were selected as the most promising upstream oncogenic miRNAs for CDK6. Some studies also found that miR‐15a‐5p is weakly expressed in various human cancers, and inhibits proliferation and metastasis of PAAD by directly targeting FGFR1 and promotes apoptosis.^25^ miR‐454‐3p has likewise been found to function in tumor metastasis and invasion of a variety of tumors.^26^, ^27^, ^28^

According to the ceRNA hypothesis, we searched for lncRNA upstream of miR‐15a‐5p and miR‐454‐3p using network database. As miR‐15a‐5p in starBase and LncBase was positively correlated with potential binding lncRNAs, it did not conform to the regulation rules of ceRNA. We only predicted upstream lncRNAs of miR‐454‐5p/CDK6 axis and 28 possible lncRNAs were identified in the intersection of starBase and LncBase. After combined expression analysis, correlation analysis, and survival analysis, HOXA11‐AS and NR2F1‐AS1 was selected as the most potential upregulated lncRNA.

It has been found that tumor immune cell infiltration can influence the therapeutic efficacy of immunotherapy and chemo‐therapy, which in turn affects the prognosis of tumor patients.^29^ In fact, immune cell infiltration plays a very important role in all stages of PC development.^30^, ^31^ For example, at the earliest premalignant states of PC, CD8+ lymphocytes accumulate in the peritumoral zone of PC, while Treg cells invade the intraepithelial region of tumor cells.^32^ As with Treg, macrophages have been found to infiltrate the cell stroma as early as during precancerous lesions.^33^ For CDK6, genetic data from systemic or conditional knockdown studies suggest that the development, maturation, or activation of many immune cells requires the involvement of cyclin‐dependent kinases (CDKs) and specific cyclins (CCNs).^34^ Deng et al. found that CDK6 significantly enhances T‐cell activation by regulating the activity of NFAT family proteins and related genes. And we likewise found that in PC, CDK6 was significantly positively correlated with NFAT4 (*R* = 0.52, *p* = 4.9 E−14), NFAT3 (*R* = 0.21, *p* = 0.003) molecules.^35^ Besides, embelin, a CDK6 inhibitor, can regulate tumor immune microenvironment and inhibit tumor growth in PC.^36^ Our study showed that CDK6 was significantly associated with multiple immune cell infiltrates and immune cell biomarkers. These findings suggest that CDK6‐mediated PC oncogenesis may be partially related to immune infiltration and that CDK6 inhibitors might be possible agents for the treatment of PC.

Notably, the effectiveness of immunotherapy is determined by the adequacy of immune checkpoint expression in the tumor tissues. We found that high CDK6 expression was correlated positively with PD‐L1, PD‐L2, or HAVCR2 expression in PC. Therefore, targeting CDK6 may increase the effectiveness of immunotherapy in PC.

In recent years, CDK4/6 inhibitors have been used in the treatment of adenocarcinoma. In addition to their classical role in inhibiting the cell cycle progression and proliferation of cancer cells, CDK4/6 inhibitors also activate the immune system by stimulating the upregulation of MHC I on the membrane of cancer cells and producing interferon (IFN) in RB‐dependent mechanism. Also, at the cellular level, CDK4/6 inhibitor treatment stimulates anti‐cancer immune responses, including reduced Treg and elevated levels of immunostimulatory molecules. In addition, the CDK4/6 inhibition also leads to upregulation of PD‐L1, suggesting that it may be clinically effective in combination with PD‐L1 blockade.^37^

Overall, we elucidated high CDK6 expression in many kinds of human cancers by bioinformatic analysis and associated with poor prognosis for pancreatic cancer. We identified a possible regulatory pathway of CDK6 in PC, namely the HOXA11‐AS/NR2F1‐AS1‐mir‐454‐3p axis. Furthermore, our current findings suggest that CDK6 may through regulating the cell cycle of tumor cells, in addition to modulating immune checkpoint expression and immune cell infiltration in tumors to exert its oncogenic effects. However, more clinical samples and more experimental studies are still need to validate our findings.

## COMPLIANCE WITH ETHICAL STANDARDS

No conflicts of interest were declared by the authors. Ethical approval of all data follows the ethical guidelines of the relevant database.

## AUTHOR CONTRIBUTIONS

**Yu‐xuan Zhao:** Data curation (equal); formal analysis (equal); investigation (equal); methodology (equal); validation (equal); visualization (equal); writing – original draft (equal); writing – review and editing (equal). **Bo‐Wen Xu:** Data curation (equal); formal analysis (equal); investigation (equal); methodology (equal); validation (equal); visualization (equal); writing – original draft (equal); writing – review and editing (equal). **Fang‐Qing Wang:** Methodology (equal); software (equal). **Feng‐Yang Jiang:** Methodology (equal); software (equal). **Jian‐wei Xu:** Conceptualization (equal); methodology (equal); resources (equal); supervision (equal); writing – review and editing (equal). **De‐xin Yu:** Conceptualization (equal); data curation (equal); methodology (equal); project administration (equal); resources (equal); supervision (equal); writing – review and editing (equal).

## FUNDING INFORMATION

This work was supported by the China national science foundation (Grant No. 81771888) and Shandong provincial natural science foundation (Grant No. ZR2017MH006).

## Supporting information


TableS1

FigureS1

FigureS2

## Data Availability

Data sharing is not applicable to this article as no new data were created or analyzed in this study.
