# Supplementary material for: nc‐RNA‐mediated high expression of CDK6 correlates with poor prognosis and immune infiltration in pancreatic cancer
Source: Cancer Med. 2022 Dec 1;12(4):5110–23. doi: 10.1002/cam4.5260 (PMC9972169; doi:10.1002/cam4.5260)
Supplement: Supplementary file 1 — TableS1 [file CAM4-12-5110-s002.docx]

S Table1. Univariate and multivariate Cox regression analysis of CDK6

| Characteristics | Total(N) | Univariate analysis | |  | Multivariate analysis | |
| --- | --- | --- | --- | --- | --- | --- |
|  |  | Hazard ratio (95% CI) | P value |  | Hazard ratio (95% CI) | P value |
| T stage | 176 |  |  |  |  |  |
| T1 | 7 | Reference |  |  |  |  |
| T2 | 24 | 1.451 (0.311-6.779) | 0.636 |  | 1.204 (0.127-11.442) | 0.871 |
| T3 | 142 | 2.742 (0.667-11.269) | 0.162 |  | 1.550 (0.126-19.090) | 0.732 |
| T4 | 3 | 1.462 (0.131-16.289) | 0.757 |  | 0.763 (0.041-14.364) | 0.857 |
| N stage | 173 |  |  |  |  |  |
| N0 | 50 | Reference |  |  |  |  |
| N1 | 123 | 2.154 (1.282-3.618) | **0.004** |  | 1.623 (0.691-3.811) | 0.267 |
| Age | 178 |  |  |  |  |  |
| <=65 | 93 | Reference |  |  |  |  |
| >65 | 85 | 1.290 (0.854-1.948) | 0.227 |  | 1.579 (0.914-2.728) | 0.101 |
| Pathologic stage | 175 |  |  |  |  |  |
| Stage I | 21 | Reference |  |  |  |  |
| Stage II | 146 | 2.333 (1.069-5.089) | **0.033** |  | 0.643 (0.123-3.354) | 0.600 |
| Stage III | 3 | 1.255 (0.153-10.275) | 0.832 |  |  |  |
| Stage IV | 5 | 1.566 (0.321-7.637) | 0.579 |  | 0.571 (0.040-8.176) | 0.680 |
| Radiation therapy | 163 |  |  |  |  |  |
| No | 118 | Reference |  |  |  |  |
| Yes | 45 | 0.508 (0.298-0.866) | **0.013** |  | 0.354 (0.171-0.732) | **0.005** |
| Primary therapy outcome | 139 |  |  |  |  |  |
| PD | 49 | Reference |  |  |  |  |
| SD | 9 | 0.544 (0.228-1.297) | 0.170 |  | 0.932 (0.323-2.690) | 0.897 |
| PR | 10 | 1.038 (0.408-2.645) | 0.937 |  | 0.852 (0.300-2.416) | 0.763 |
| CR | 71 | 0.335 (0.201-0.559) | **<0.001** |  | 0.553 (0.302-1.013) | 0.055 |
| Residual tumor | 164 |  |  |  |  |  |
| R0 | 107 | Reference |  |  |  |  |
| R1 | 52 | 1.710 (1.089-2.684) | **0.020** |  | 1.713 (0.898-3.269) | 0.103 |
| R2 | 5 | 1.035 (0.251-4.261) | 0.962 |  | 1.016 (0.204-5.066) | 0.985 |
| Histologic grade | 176 |  |  |  |  |  |
| G1 | 31 | Reference |  |  |  |  |
| G2 | 95 | 1.959 (1.007-3.808) | **0.048** |  | 2.120 (0.833-5.397) | 0.115 |
| G3 | 48 | 2.625 (1.304-5.283) | **0.007** |  | 3.149 (1.127-8.801) | **0.029** |
| G4 | 2 | 1.651 (0.211-12.893) | 0.632 |  | 3.157 (0.264-37.817) | 0.364 |
| Anatomic neoplasm subdivision | 178 |  |  |  |  |  |
| Head of Pancreas | 138 | Reference |  |  |  |  |
| Other | 40 | 0.417 (0.231-0.754) | **0.004** |  | 0.504 (0.223-1.141) | 0.100 |
| CDK6 | 178 | 1.860 (1.411-2.451) | **<0.001** |  | 1.966 (1.215-3.181) | **0.006** |
| M stage | 84 |  |  |  |  |  |
| M0 | 79 | Reference |  |  |  |  |
| M1 | 5 | 0.756 (0.181-3.157) | 0.701 |  |  |  |
| Gender | 178 |  |  |  |  |  |
| Female | 80 | Reference |  |  |  |  |
| Male | 98 | 0.809 (0.537-1.219) | 0.311 |  |  |  |
| Smoker | 144 |  |  |  |  |  |
| No | 65 | Reference |  |  |  |  |
| Yes | 79 | 1.086 (0.687-1.719) | 0.724 |  |  |  |
| History of diabetes | 146 |  |  |  |  |  |
| No | 108 | Reference |  |  |  |  |
| Yes | 38 | 0.927 (0.532-1.615) | 0.790 |  |  |  |
| History of chronic pancreatitis | 141 |  |  |  |  |  |
| No | 128 | Reference |  |  |  |  |
| Yes | 13 | 1.177 (0.562-2.464) | 0.666 |  |  |  |
| Race | 174 |  |  |  |  |  |
| Asian | 11 | Reference |  |  |  |  |
| Black or African American | 6 | 1.205 (0.323-4.503) | 0.781 |  |  |  |
| White | 157 | 1.256 (0.508-3.109) | 0.622 |  |  |  |
| Alcohol history | 166 |  |  |  |  |  |
| No | 65 | Reference |  |  |  |  |
| Yes | 101 | 1.147 (0.738-1.783) | 0.542 |  |  |  |
| Family history of cancer | 110 |  |  |  |  |  |
| No | 47 | Reference |  |  |  |  |
| Yes | 63 | 1.117 (0.650-1.920) | 0.689 |  |  |  |
