# Supplementary figures and images for: nc‐RNA‐mediated high expression of CDK6 correlates with poor prognosis and immune infiltration in pancreatic cancer
Source: Cancer Med. 2022 Dec 1;12(4):5110–23. doi: 10.1002/cam4.5260 (PMC9972169; doi:10.1002/cam4.5260)

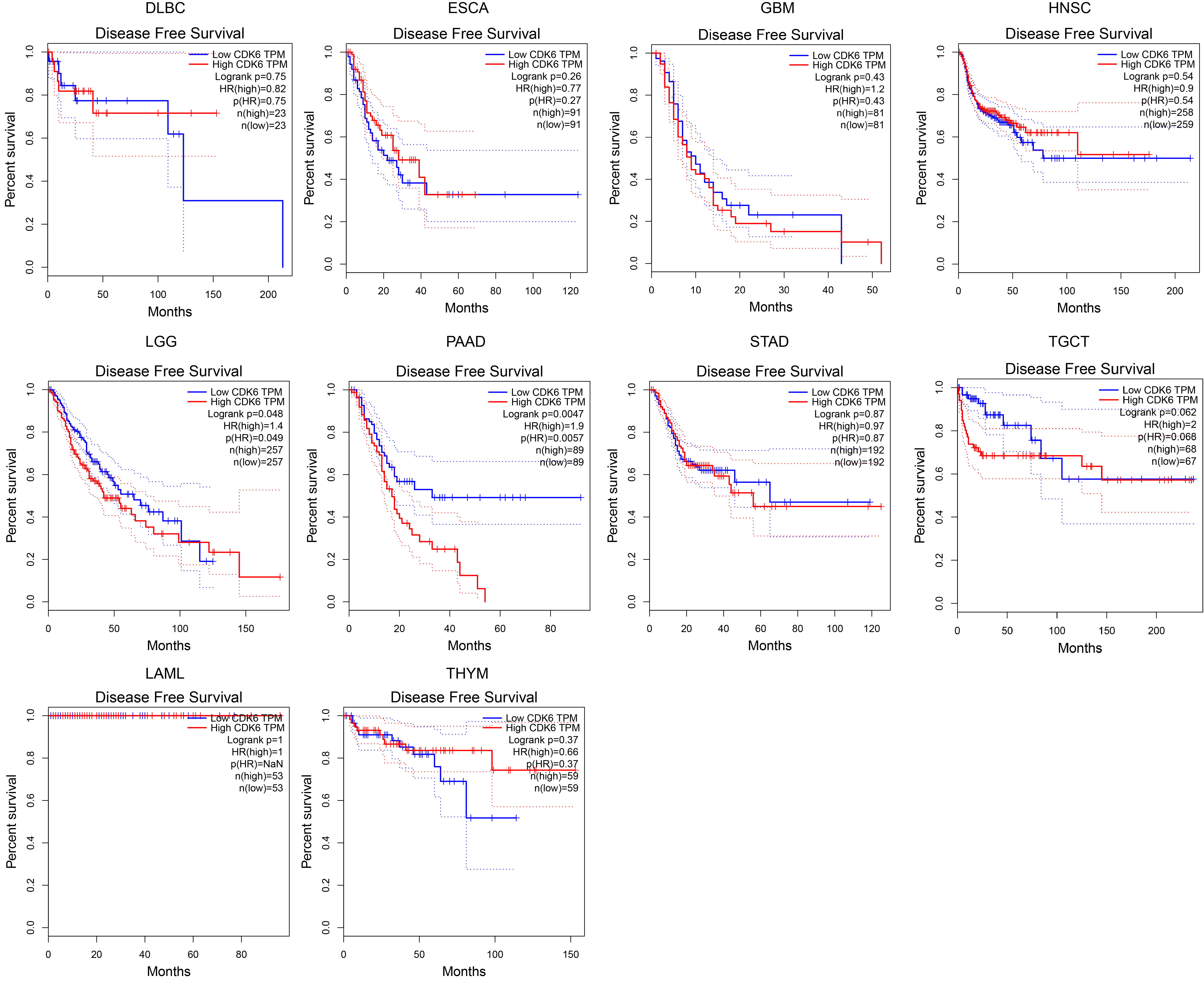

Supplement: Supplementary file 2 — FigureS1 [file CAM4-12-5110-s001.tif]

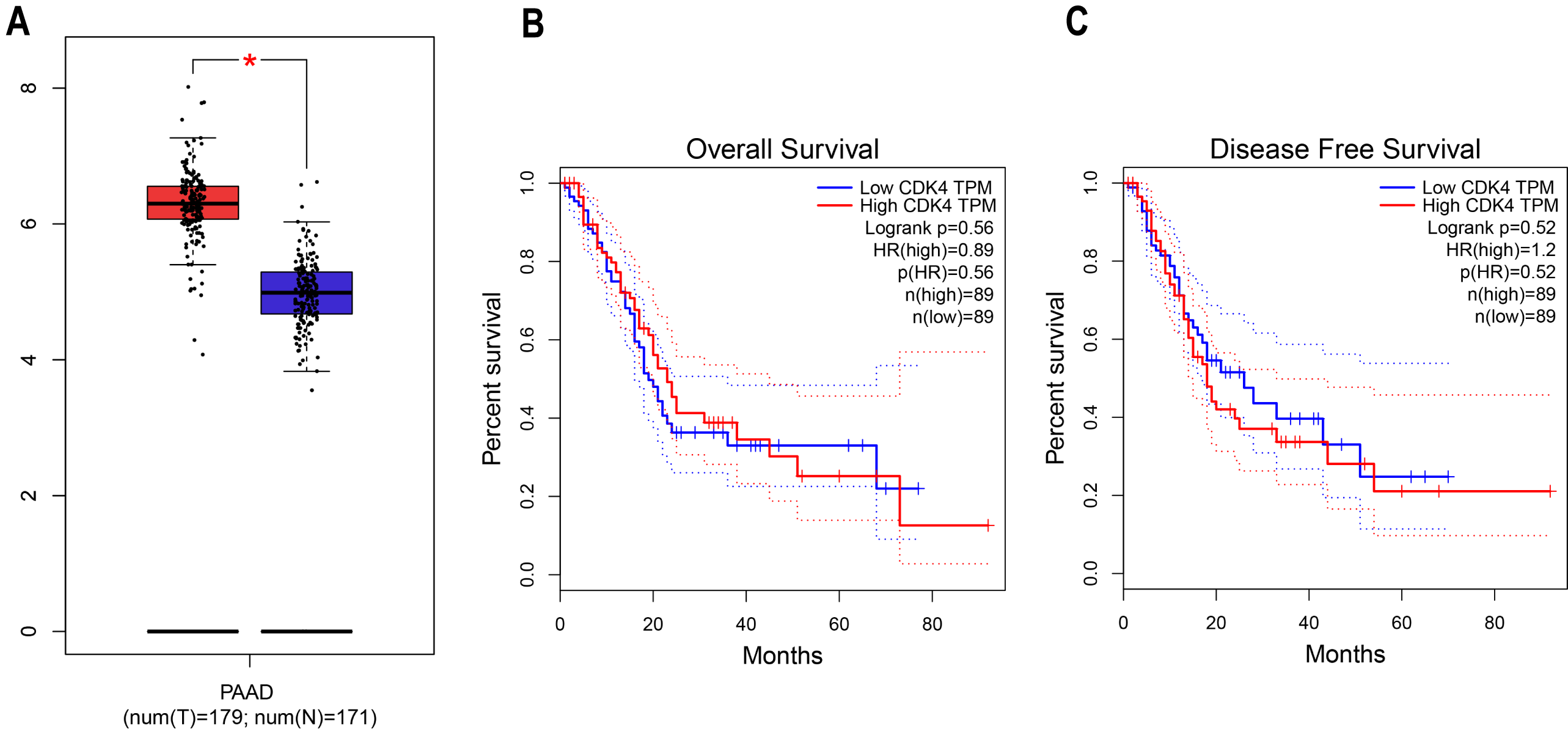

Supplement: Supplementary file 3 — FigureS2 [file CAM4-12-5110-s003.tif]
